# Advances in Understanding and Managing Chronic Urticaria

**DOI:** 10.12688/f1000research.7246.1

**Published:** 2016-02-16

**Authors:** Yasmin Moolani, Charles Lynde, Gordon Sussman

**Affiliations:** 1 Division of Clinical Immunology and Allergy, University of Toronto, Toronto, OH, Canada; 2 Division of Dermatology, University of Toronto, Toronto, ON, Canada; 3 Lynderm Research, Lynde Institute for Dermatology, Markham, ON, Canada; 4 Gordon Sussman Clinical Research Inc, GSCR, Toronto, ON, Canada

**Keywords:** Chronic Urticaria, Classification, Management, Antihistamines, Up-dosing, Omalizumab

## Abstract

There have been recent advances in the classification and management of chronic urticaria. The new term chronic spontaneous urticaria (CSU) has replaced chronic idiopathic urticaria and chronic autoimmune urticaria. In addition, chronic inducible urticaria (CINDU) has replaced physical urticaria and includes other forms of inducible urticaria, such as cholinergic and aquagenic urticaria. Furthermore, novel research has resulted in a new understanding with guidelines being revised in the past year by both the American Academy of Allergy, Asthma, and Immunology (AAAAI) and the European Academy of Allergy and Clinical Immunology (EAACI)/Global Allergy and Asthma European Network (GA ^2^LEN)/European Dermatology Forum (EDF)/World Allergy Organization (WAO). There are some differences in the recommendations, which will be discussed, but the core updates are common to both groups. The basic treatment for chronic urticaria involves second-generation non-sedating non-impairing H _1_ antihistamines as first-line treatment. This is followed by up to a 4-fold increase in the licensed dose of these H _1_ antihistamines. The major therapeutic advance in recent years has been in third-line treatment with omalizumab, a humanized monoclonal anti-immunoglobulin E (anti-IgE) antibody that prevents binding of IgE to the high-affinity IgE receptor. Several multicenter randomized controlled trials have shown safety and efficacy of omalizumab for CSU. There are also some small studies showing efficacy of omalizumab in CINDU. While there were previously many treatment options which were lacking in strong evidence, we are moving into an era where the treatment algorithm for chronic urticaria is simplified and contains more evidence-based, effective, and less toxic treatment options.

## Introduction

Urticaria (hives) is a relatively common condition, with a point prevalence of about 0.5–1% ^1^. The peak incidence is in the range of 20–40 years. Urticaria is the general term for a cutaneous response characterized by wheals and swellings. A deeper localized swelling often associated with urticaria is called angioedema. Urticaria is mediated by mast cell degranulation. Mast cells can be activated by immunologic and non-immunologic mechanisms, which lead to degranulation of inflammatory mediators including histamine, leukotrienes, and prostaglandins. Release of these mediators causes the characteristic pruritus, vascular permeability, and edema.

## Classification

**Figure 1.**
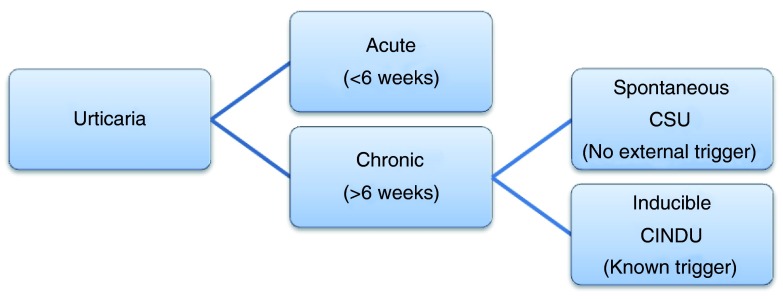
Classification of Urticaria. The revised classification of urticaria divides chronic urticaria into chronic spontaneous urticaria (CSU), with no external trigger, and chronic inducible urticaria (CINDU), with an identifiable trigger. Acute urticaria remains as that which lasts less than 6 weeks.

Acute urticaria is defined as hives that last less than 6 weeks, while chronic urticaria refers to urticaria that occurs intermittently for at least 6 weeks, typically on most days of the week. Acute urticaria can occur spontaneously or in response to a trigger. Triggers of acute urticaria are typically acute viral infections or allergic reactions to foods, medications, latex, or insects. Chronic urticaria can also be spontaneous or inducible, though the triggers of inducible urticaria are different compared to those of acute urticaria. Inducible urticaria involves hiving responses resulting from physical stimuli including scratch (dermographism) and cold contact urticaria. Additional triggers of chronic urticaria include pressure, temperature change, sun exposure, water exposure, and exercise ( Figure 1, Table 1) ^2– 5^.

**Table 1.** Triggers of Acute Urticaria and Chronic Inducible Urticaria. The triggers of acute and chronic urticaria are different and are outlined in this table.

*Classification*	*Acute Urticaria* *(<6 weeks)*	*Chronic Inducible Urticaria* *(>6 weeks)*
*Triggers*	Infection	Dermographism
	Food	Cholinergic
	Medication	Cold-induced
	Venom	Solar
	Latex	Aquagenic
	Contact	Vibration
	Idiopathic	Delayed-pressure

## New terminology

While the pathogenesis of urticaria remains incompletely understood, our knowledge of the etiology of chronic urticaria has increased. We are able to differentiate the older terminology of chronic autoimmune urticaria or chronic idiopathic urticaria from physically induced urticaria ^6^. However, now physical urticaria has been revised to chronic inducible urticaria (CINDU) to reflect the external trigger and inducible nature. The term CINDU also includes cholinergic, aquagenic, and contact urticaria. The remaining forms of urticaria, which occur without an external trigger and instead via an endogenous mechanism, are classified as chronic spontaneous urticaria (CSU). This is simplified and more specific than the term chronic idiopathic urticaria, which is now falling out of use ^2^ ( Figure 1).

## Assessing disease severity and impact on quality of life

The primary symptom of urticaria is pruritus. Urticaria generally causes significant disability adversely affecting an individual’s life. New tools have been developed to quantify the effect of urticaria on quality of life. One such tool is the Urticaria Activity Score (UAS7), which is a validated score recommended by urticaria guidelines ^7^. In the UAS7, the patient records the severity of itching and the number of wheals daily for 7 days. A score of <7 in 1 week indicates control of disease, whereas a score of >28 per week indicates severe disease. This tool allows for efficient clinical practice, maximizing the information gathered during patient visits while minimizing the use of resources and time. While the UAS7 is a prospective tool, a retrospective tool was also developed, the Urticaria Control Test (UCT) ^8^. Additional questionnaires have been developed and are recommended for use to assess the impact of urticaria on quality of life. The recently developed tools include the Chronic Urticaria Quality of Life Questionnaire (CU-Q2oL) ^9^ and the Urticaria Severity Score ^10^. These tools were designed specifically for urticaria since other more generic questionnaires do not capture the nuances of the impact of urticaria on one’s quality of life ( Table 2).

**Table 2.** Tools to assess the impact of chronic urticaria on quality of life. There are a number of recently developed tools specifically for urticaria to assess severity and impact on quality of life.

Tool	Description
Urticaria Activity Score (UAS7)	Prospective tool, patients record severity of itch and number of wheals daily, must be recorded by patient for 7 consecutive days, assesses disease activity ^7^
Urticaria Control Test (UCT)	Retrospective tool, four-item questionnaire to determine control of disease ^8^
Chronic Urticaria Quality of Life Questionnaire (CU-Q2oL)	Retrospective tool, 23-item questionnaire, assesses quality of life ^9^
Urticaria Severity Score (USS)	Instrument for monitoring severity, 12 questions ^10^

## Advances in management

Recently, new guidelines have been published by the American Academy of Allergy, Asthma, and Immunology (AAAAI) and the European Academy of Allergy and Clinical Immunology (EAACI)/Global Allergy and Asthma European Network (GA ^2^LEN)/European Dermatology Forum (EDF)/World Allergy Organization (WAO) that update what is currently understood about urticaria ^3– 5^. There are several other published regional guidelines with similar recommendations, including Canadian ^11^. With the exception of the American guidelines, the other organizations put forth a similar algorithm. That is, the following stepwise approach: first-line treatment includes second-generation H _1_ antihistamines, second-line therapy involves up-dosing the second-generation H _1_ antihistamine, and third-line treatment includes a new medication, omalizumab, which we recommend to be used before a more toxic medication, cyclosporine A.

One of the notable differences in the guidelines put forth by the American versus European/World guidelines is the role for first-generation H _1_ antihistamines. The AAAAI keeps first-generation H _1_ antihistamines in the treatment algorithm. The EAACI/GA ^2^LEN/EDF/WAO guidelines specifically recommend avoiding first-generation H _1_ antihistamines based on the benefit-to-risk ratio of these agents. The known risks are cited as well as the high underestimated risk potential when these agents are taken in the evening, such as changes in REM sleep patterns and impairment of cognitive functions ^12^. For similar reasons, the EAACI/GA ^2^LEN/EDF/WAO guidelines do not address a role for hydroxyzine or doxepin, whereas the AAAAI guidelines include these medications as options if up-dosing of non-sedating, non-impairing H _1_ antihistamines is not successful at complete control of symptoms.

The EAACI/GA ^2^LEN/EDF/WAO guidelines do not include H _2_ antihistamines in their algorithm due to a recent Cochrane review that shows lack of evidence of efficacy of these medications ^13^. Thus, H _2_ antihistamines are advised only on an individual case basis but not as first-, second- or third-line treatment. The AAAAI guidelines, however, consider the options of up-dosing second-generation H _1_ antihistamines, adding other second-generation H _1_ antihistamines, and adding H _2_ antagonists, leukotriene receptor antagonists or first-generation H _1_ antihistamines at bedtime to all be equally weighted second-line options ^3^. In doing this, however, the adverse reaction profile would increase.

Both guidelines recommend that corticosteroids should be considered only for the short-term intervention and avoided as long-term treatments due to the significant number of side effects and alternative options for treatment. Both guidelines also acknowledge a role for cyclosporine A as an add-on treatment for patients who have refractory chronic urticaria that is not responsive to the above mentioned treatments ^3, 5^.

Both guidelines have also incorporated a new third-line treatment option, omalizumab, a subcutaneously injected, biologic medication ^3, 5^. There is a large body of new data showing efficacy of omalizumab in various subtypes of chronic urticaria with a relatively low level of adverse effects ^18– 24^. Omalizumab is a recombinant humanized immunioglobulin G1 (IgG1) monoclonal antibody that binds to IgE. In binding IgE, omalizumab inhibits binding of IgE to the high-affinity IgE receptor (FcεRI) found on the surface of mast cells and basophils. By inhibiting binding of IgE to the receptor, the release of inflammatory mediators such as histamine, leukotrienes, and prostaglandins is limited, thus blunting the inflammatory response ^14^. By inhibiting IgE from binding the FcεRI receptor, omalizumab also has the effect of down-regulating the FcεRI receptor on the surface of mast cells and basophils. This monoclonal antibody has also been shown to decrease the release of circulating interleukin-6 and tumor necrosis factor α and to decrease the recruitment of T cells, eosinophils, and macrophages in the inflammatory response ^15^. The overall effect of omalizumab is to prevent urticaria and angioedema ^14^.

Omalizumab was approved in Europe and North America in 2014 for the treatment of CSU in adults and adolescents 12 years of age and older who do not respond to H _1_ antihistamines ^16, 17^. This approval was based on three phase III randomized clinical trials evaluating over 1000 patients’ treatment response with inadequately controlled CSU ^18– 20^. There have also been several published real life trials demonstrating efficacy and safety ^21– 23^. Additionally, there are smaller case studies assessing the efficacy of omalizumab in various subtypes of CINDU, and while the number of overall cases is low, all have shown notable improvement in urticaria symptoms ^24^. Future research is, therefore, required to evaluate the role of omalizumab in the various subtypes of chronic urticaria as well as to establish standardized protocols for dosing and monitoring adverse effects of long-term therapy.

## Conclusion

A number of notable changes have occurred recently in the guidelines for managing chronic urticaria. Overall, the classification and management have become more simplified. There are new tools to track and monitor the quality of life of patients with this challenging disease. Furthermore, the recent addition of omalizumab to the treatment algorithm for CSU has offered a more effective and less toxic treatment option to those suffering with refractory disease to older treatment strategies. With the many trials currently underway for CINDU, the outlook is optimistic.
